# A Method to Measure Hydrolytic Activity of Adenosinetriphosphatases (ATPases)

**DOI:** 10.1371/journal.pone.0058615

**Published:** 2013-03-05

**Authors:** Gianluca Bartolommei, Maria Rosa Moncelli, Francesco Tadini-Buoninsegni

**Affiliations:** Department of Chemistry “Ugo Schiff”, University of Florence, Sesto Fiorentino, Italy; University of Leeds, United Kingdom

## Abstract

The detection of small amounts (nanomoles) of inorganic phosphate has a great interest in biochemistry. In particular, phosphate detection is useful to evaluate the rate of hydrolysis of phosphatases, that are enzymes able to remove phosphate from their substrate by hydrolytic cleavage. The hydrolysis rate is correlated to enzyme activity, an extremely important functional parameter.

Among phosphatases there are the cation transporting adenosinetriphosphatases (ATPases), that produce inorganic phosphate by cleavage of the γ-phosphate of ATP. These membrane transporters have many fundamental physiological roles and are emerging as potential drug targets. ATPase hydrolytic activity is measured to test enzyme functionality, but it also provides useful information on possible inhibitory effects of molecules that interfere with the hydrolytic process.

We have optimized a molybdenum-based protocol that makes use of potassium antimony (III) oxide tartrate (originally employed for phosphate detection in environmental analysis) to allow its use with phosphatase enzymes. In particular, the method was successfully applied to native and recombinant ATPases to demonstrate its reliability, validity, sensitivity and versatility. Our method introduces significant improvements to well-established experimental assays, which are currently employed for ATPase activity measurements. Therefore, it may be valuable in biochemical and biomedical investigations of ATPase enzymes, in combination with more specific tests, as well as in high throughput drug screening.

## Introduction

Adenosinetriphosphatases (ATPases) are enzymes that produce inorganic phosphate (Pi) by cleavage of the γ-phosphate of ATP. Main representative members of this large family are the cation-transport ATPases, e.g. sarcoplasmic reticulum Ca-ATPase (SERCA) [1], [2] and Na,K-ATPase [3], [4]. These proteins couple ATP hydrolysis to the transport of ionic species against their electrochemical potential gradient.

Ion translocation is normally coupled to ATP hydrolysis through a cyclic sequence of chemical reactions denoted as “enzymatic cycle”. The enzymatic cycle includes initial enzyme activation triggered by cation binding, followed by ATP utilization to form a phosphorylated intermediate. The free energy derived from ATP is then utilized by the phosphoenzyme for a conformational transition, that favors displacement and release of the bound cation. Binding of counter-transported ions induces dephosphorylation of the enzyme, followed by release of the counterions during a conformational transition to the initial state [5]. Therefore, Pi detection is useful to evaluate the rate of Pi production by ATPases and the related enzyme activity, an extremely important functional parameter.

Phosphate detection is fundamental in environmental analysis too. In particular, phosphate is an important routine parameter in water analysis, being simultaneously an essential macronutrient and a possible pollutant, when its concentration is abnormally high. The quantification of phosphate in different water bodies is important since an increase in phosphate concentration in surface waters is usually linked to diffuse sources [6]. On the other hand, phosphorus determination in soil samples provides important information on phosphorus availability for plants [7].

Due to the broad relevance of phosphorus, different phosphate detection methods have been optimized during years. These methods are usually based on the chemistry of molybdenum. In fact, it is well known that phosphate and molybdic acid form a complex that can be reduced to produce a deep-blue-colored complex called molybdenum blue [8]. Classical experimental protocols for Pi detection involve the use of ammonium heptamolybdate in acid environment (HCl or H_2_SO_4_), together with a reducing agent such as sodium sulfite [9], stannous chloride [10], phenylhydrazine [11], aminonaphtholsulfonic acid [9], ascorbic acid [12], ρ-methylaminophenolsulfate [13], N-phenyl-ρ-phenylenediamine [14] or ferrous sulfate [15]. The choice of the reducing agent is critical for determining the stability of the reduced complex and, moreover, affects the spectroscopic properties of the produced molybdenum blue species [16].

A modified protocol involving potassium antimony (III) oxide tartrate as an additional reagent exists. The use of this compound has been described in environmental analysis on soil samples [7], [17] or water [6], [18], but never in enzymology. Potassium antimony (III) oxide tartrate reacts with ammonium heptamolybdate in an acid medium with diluted solutions of phosphate to form an antimony-phosphomolybdate complex. This complex can be reduced to an intensely blue-colored complex by one of the reducing agents mentioned above [7], [18].

This paper presents, for the first time, the application of the method based on the formation of the antimony-phosphomolybdate complex to the determination of the hydrolytic activity of ATPases. Following an optimization of the experimental protocol, the method was applied to native and recombinant ATPases to demonstrate its validity, sensitivity and versatility.

## Materials and Methods

### Chemicals

Sodium azide (extra-pure), sulfuric acid (95–97%, pro-analysi), ammonium heptamolybdate tetrahydrate (pro-analysi), tri-sodium citrate dihydrate (pro-analysi), MgCl_2_ hexahydrate (pro-analysi), tris(hydroxymethyl)aminomethane (TRIS; pro-analysi), potassium antimony (III) oxide tartrate trihydrate (extra pure), KH_2_PO_4_ (suprapur), KCl (suprapur), NaCl (suprapur) and CaCl_2_ (suprapur) were purchased from Merck. L(+)-ascorbic acid (normapur) was from VWR BDH Prolabo. Na_2_-ATP hydrate and ouabain were purchased from Fluka. Thapsigargin (TG), calcium ionophore A23187 (calcimycin), ethylene glycol-bis(2-aminoethylether)-N,N,N',N'-tetraacetic acid (EGTA; 97%, for Molecular Biology) and 3-(N-morpholino)propanesulfonic acid (MOPS) were obtained from Sigma.

The water used for the preparation of all solutions was produced by a purification system (Millipore, Direct-Q 5), that eliminates bacterial content through a 0.22 µm sterile filter (Millipak 40), reduces Total Organic Carbon content to less than 10 µg/l (Quantum EX Ultrapure Organex Cartridge) and lowers ionic concentration, thus increasing resistivity to a maximum value of 18.2 MΩ·cm (Progard 2 Pre-treatment pack).

### Ethics Statement

Isolation of proteins from rabbit was performed in the Department of Pharmacology, University of Florence, Italy. Animal manipulations were carried out according to the Italian Guidelines for Animal Care (DL 116/92, application of the European Communities Council Directive 86/609/EEC) and approved by the local IACUC (Advisory Committee for Ethical and Juridical Control of the Center for Housing of Laboratory Animals of the University of Florence). All efforts were made to minimize animal sufferings.

### Protein preparation

SERCA was isolated from rabbit hind-leg skeletal muscle in the form of native vesicles according to [19]. The obtained vesicles are not permeable to calcium ions. The total protein concentration, determined by the Lowry procedure [20], was 8.4 mg/ml.

Membrane fragments containing the Na,K-ATPase were isolated from rabbit kidneys following the procedure C described in [21]. In this case, the total protein concentration was 2.07 mg/ml.

Recombinant SERCA (WT and D351N mutant) was expressed in COS-1 cells using adenoviral vectors as previously described [22]. Total protein concentration was 2.6 mg/ml for the WT enzyme (78 µg/ml of SERCA, corresponding to 3%) and 11.8 mg/ml for the D351N mutant (0.80 mg/ml of SERCA, corresponding to about 7%). The content of expressed SERCA was evaluated by SDS gel electrophoresis and Western blotting [22].

### Coloring solution

The coloring solution employed for Pi determination was composed of sulfuric acid, ascorbic acid, ammonium heptamolybdate and potassium antimony (III) oxide tartrate (herein indicated as “tartrate”). These compounds produce an antimony-molybdate complex that converts in antimony-phosphomolybdate when phosphorus is present as inorganic (orto)phosphate ion [7], [18]. The latter complex is blue-colored and therefore adsorbs light in the visible range, allowing its use in quantitative analysis.

Solutions were always prepared from the following concentrated stocks: 2.5 M H_2_SO_4_, 0.3 M ascorbic acid, 4 mM tartrate and 24 mM ammonium heptamolybdate.

### Measurement of enzymatic activity

The enzyme (native or recombinant) was incubated (FALC Thermoblock) in a buffer solution at 37°C and the reaction was started by addition of 1 mM ATP. For SERCA, the buffer solution contained 80 mM KCl, 25 mM MOPS (pH 7.0 by TRIS), 3 mM MgCl_2_, 5 mM sodium azide, 0.2 mM EGTA, 0.2 mM CaCl_2_ (about 10 µM free calcium) and 2 µM A23187. In the case of Na,K-ATPase, the buffer solution was composed by 20 mM KCl, 100 mM NaCl, 25 mM MOPS (pH 7.0 by TRIS) and 3 mM MgCl_2_. Blank samples were prepared in the absence of calcium ions and in the presence of 2 mM EGTA (SERCA) or in the absence of sodium ions (Na,K-ATPase). Control experiments were performed in the presence of 1 µM thapsigargin (SERCA) or 50 µM ouabain (Na,K-ATPase). The total protein concentration was 10 µg/mL for native and recombinant (WT) SERCA, 1 µg/mL for Na,K-ATPase and 4.5 µg/mL for mutant (D351N) SERCA.

After incubation, aliquots were taken at subsequent times and immediately added to the coloring solution contained in glass disposable test tubes (Corning, mod. 99445-12). Usually, aliquots of 100 µL were added to 900 µL of coloring solution, giving a total final volume of 1 mL. This addition suddenly interrupts the enzymatic ATP hydrolysis, and, therefore, Pi release, due to enzyme denaturation produced by the strong acid conditions.

For each protein, five aliquots, each as triplicate, were taken at different times to plot an activity curve (Pi vs time). Blank was also evaluated as a triplicate. Where indicated, sodium citrate was added from a 10% (w/w) stock 10 minutes after aliquot addition.

The calibration curve was determined by preparing standard solutions of Pi from 1 or 0.1 mM aqueous stock solutions.

All measurements were carried out by an UV/Vis spectrophotometer (Jasco, mod. V-560) provided with a stirring and thermostatable holder (Jasco, mod. EHC-477S). The bandwidth was settled to 2 nm. Acquisition data pitch was 1 s for kinetic measurements and 0.5 nm for spectra registration. Quartz semimicro cuvettes (Hellma, 109.004F-QS) were used to allow continuous stirring of the solution. The temperature was always maintained at 20°C by a Peltier temperature controller (Jasco, mod. EHC-477T).

### Statistics

UV/Vis spectra were reproducible with a standard deviation of less than 2%.

For activity measurements, error bars shown in figures indicate standard deviation of at least three measurements for a representative experiment. Where indicated, errors were expressed as standard error of the mean (SEM) of a number of independent measurements.

## Results

In the present work, the optimal experimental conditions (concentration of reagents and detection wavelength) for absorbance measurements were first determined. The method was then tested on different ATPase enzymes, both in native and recombinant form.

### Optimization of the experimental conditions

To determine the optimal concentrations of reagents in the coloring solution, the formation of the antimony-phosphomolybdate complex and its spectroscopic properties were examined by varying the concentration of a single reagent and maintaining constant that of the other three. Moreover, the amount of Pi was kept constant at 15 nmol for all experiments.

We started by considering the dependence on sulfuric acid concentration. The reaction mixture contained 0.48 mM ammonium-heptamolybdate, 6 mM ascorbic acid and 40 µM tartrate, plus the desired amount of H_2_SO_4_. The reaction was started by addition of Pi to the cuvette under continuous stirring at 20°C. Color development, indicating complex formation, was followed by measuring the absorbance at 710 nm as a function of time.

**Figure 1:**
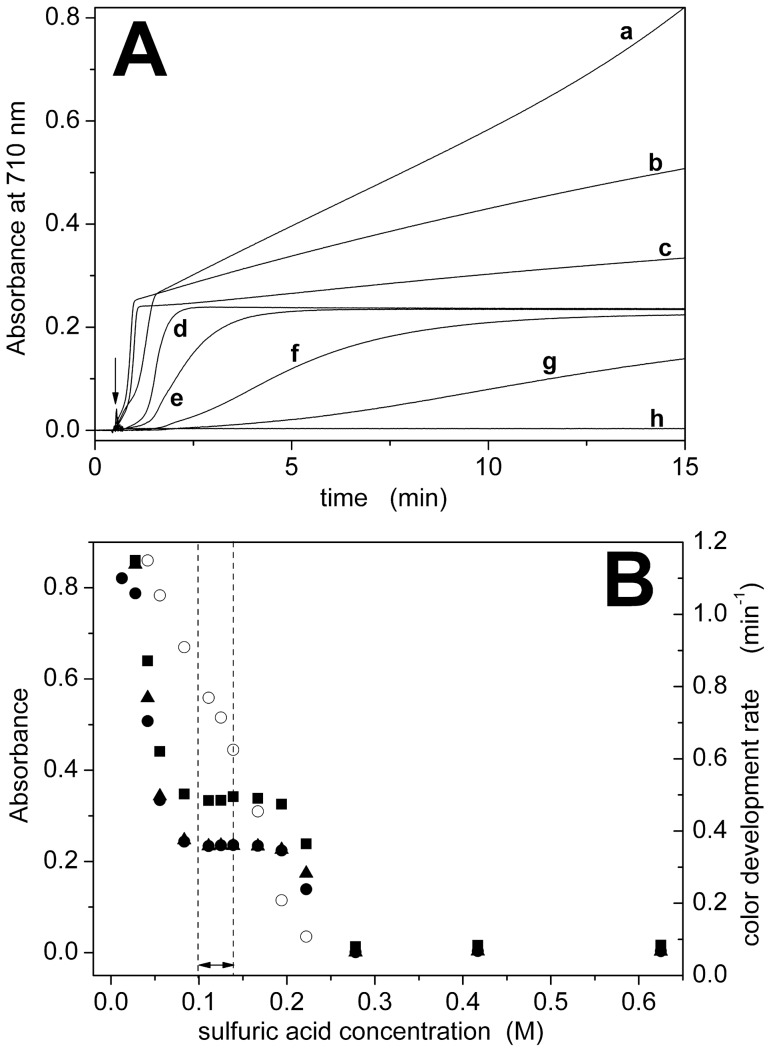
Dependence on H_2_SO_4_ concentration. (A) Color development curves determined at 710 nm for different concentrations of sulfuric acid: 12.5 mM (a), 41.7 mM (b), 55.6 mM (c), 139 mM (d), 167 mM (e), 194 mM (f), 222 mM (g) and 625 mM (h). The arrow indicates the addition of 15 nmol of Pi to the cuvette. (B) Dependence of absorbance (•, ▴, ▪) or color development rate (○) on acid concentration. Absorbance data were taken from the color development curves at t = 15 min (•) or from the visible spectra (Fig. S1) at 710 nm (▴) or 890 nm (▪). The dashed lines indicate the optimal concentration range (see also Table 1).

Fig. 1A shows representative color-development curves for different H_2_SO_4_ concentrations, whereas Fig. S1 shows the corresponding visible spectra between 400 and 900 nm taken 15 minutes after addition of Pi. We considered a concentration range from 12.5 to 625 mM H_2_SO_4_ (Table 1)_._ The color developed more slowly when the acid concentration was higher, whereas a faster initial color development was observed by decreasing acid concentration (Fig. 1A and B). Color development rates were determined as the reciprocal of the time needed to reach half of the stationary absorbance value. Below 80 mM H_2_SO_4_ a stationary value of the absorbance could not be obtained (Fig. 1A). Therefore, the optimal range of H_2_SO_4_ concentration for fast and stable color development was 100–140 mM (Fig. 1B and Table 1).

**Table 1:** Optimization of experimental conditions.

Reagent	Solution composition	Concentration range	Optimal concentration range
H_2_SO_4_	0.48 mM ammonium-heptamolybdate	12.5–625 mM	100–140 mM
	6 mM ascorbic acid		
	40 µM tartrate		
Ammonium heptamolybdate	125 mM H_2_SO_4_	0.048–4.8 mM	0.35–0.70 mM
	6 mM ascorbic acid		
	40 µM tartrate		
Ascorbic acid	125 mM H_2_SO_4_	0.1–100 mM	>3 mM
	0.48 mM ammonium heptamolybdate		
	40 µM tartrate		
Tartrate	125 mM H_2_SO_4_	0.88–400 µM	20–100 µM
	0.48 mM ammonium heptamolybdate		
	6 mM ascorbic acid		

Experimental conditions employed during the optimization procedure.

Similar experiments were carried out by varying the concentrations of the other three reagents one by one within a certain concentration range according to Table 1. For each reagent, an optimal concentration range was found.

**Figure 2:**
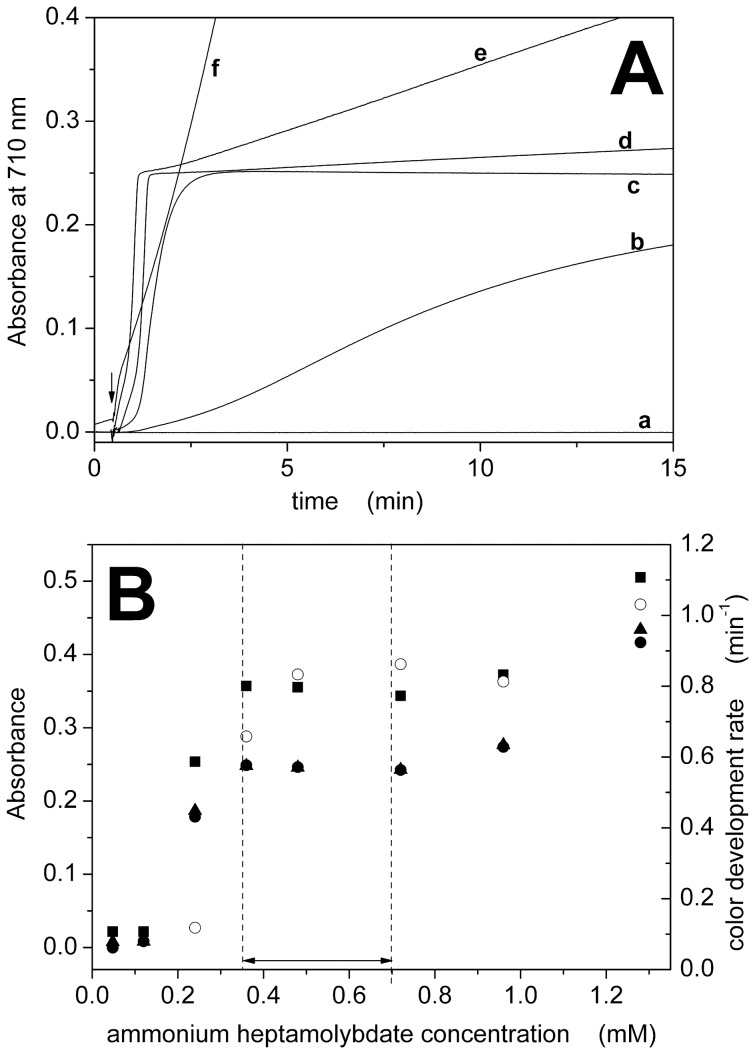
Dependence on ammonium heptamolybdate concentration. (A) Color development curves measured at 710 nm in the presence of different concentrations of ammonium molybdate: 0.048 mM (a), 0.24 mM (b), 0.36 mM (c), 0.96 mM (d), 1.28 mM (e) and 4.8 mM (f). Inorganic phosphate (15 nmol) was added where indicated by the arrow. (B) Dependence of absorbance (•, ▴, ▪) or color development rate (○) on ammonium heptamolybdate concentration. Absorbance data were taken from the development curves at t = 15 min (•) or from the visible spectra (Fig. S2) at 710 nm (▴) and 890 nm (▪). The optimal concentration range is indicated by the dashed lines (Table 1).

By varying the concentration of ammonium heptamolybdate, we observed that color developed very slowly or not developed at all at molybdate concentrations lower than 0.35 mM (Fig. 2A and 2B). On the contrary, color developed rapidly but without attaining a stable absorbance value at concentrations higher than 0.70 mM (Fig. 2A). Therefore, the optimal concentration range for ammonium molybdate was 0.35–0.70 mM (Fig. 2B and Table 1). Representative visible spectra between 400 and 900 nm are reported as (Fig. S2).

**Figure 3:**
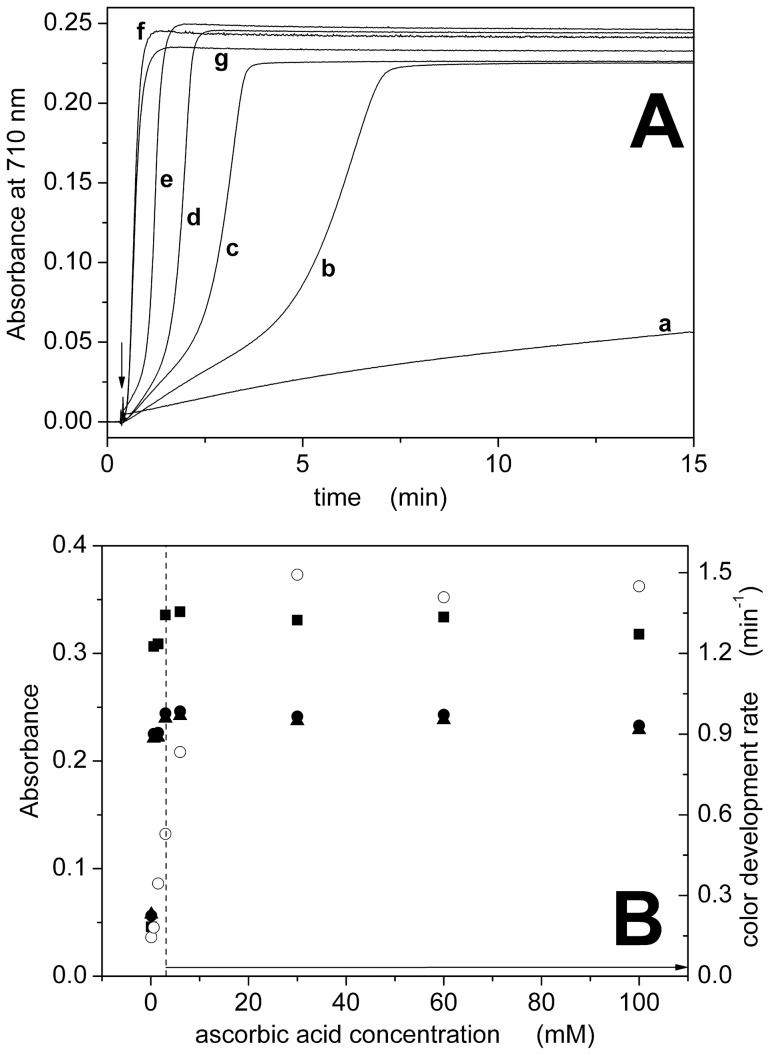
Dependence on ascorbic acid concentration. (A) Color development curves for different concentrations of ascorbic acid at 710 nm: 0.1 mM (a), 0.6 mM (b), 1.5 mM (c), 3 mM (d), 6 mM (e), 30 mM (f) and 100 mM (g). The arrow indicates addition of 15 nmol of Pi to the cuvette to start the reaction. (B) Dependence of absorbance (•, ▴, ▪) or color development rate (○) on acid concentration. Absorbance data were taken from the color development curves at t = 15 min (•) or from the visible spectra at 710 nm (▴) and 890 nm (▪) (Fig. S3). The dashed and the arrowed line indicate the optimal concentration range (Table 1).

Ascorbic acid is the reducing agent that is required for the formation of the molybden-blue complex. Therefore, color development should be slower and/or not complete by decreasing ascorbic acid concentration. This was confirmed in our experiments, as reported in Fig. 3A. On the other hand, color development becomes independent on ascorbic acid at concentrations greater than 3 mM and remains fast and stable in time (Fig. 3A and 3B). Examples of acquired visible spectra between 400 and 900 nm are reported as (Fig. S3). Ascorbic acid should then be present at concentrations greater than 3 mM (Fig. 3B and Table 1).

**Figure 4:**
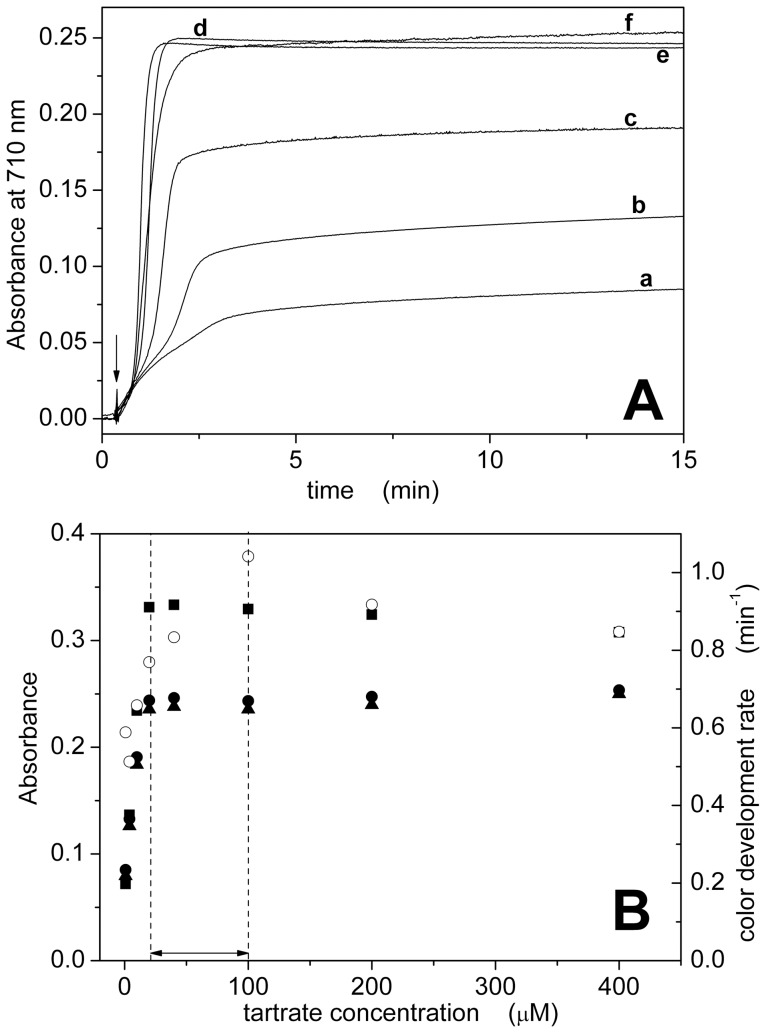
Dependence on potassium-antimony (III) oxide tartrate concentration. (A) Color development curves acquired at 710 nm for different concentrations of tartrate: 0.88 µM (a), 4 µM (b), 10 µM (c), 40 µM (d), 100 µM (e) and 400 µM (f). The reaction was started by the addition of 15 nmol of Pi (indicated by the arrow). (B) Dependence of absorbance (•, ▴, ▪) or color development rate (○) on tartrate concentration. Absorbance data were taken from the development curves at t = 15 min (•) or from the visible spectra (Fig. S4) at 710 nm (▴) and 890 nm (▪). The optimal concentration range is indicated by the dashed lines (see also Table 1).

Finally, tartrate concentration was varied in the range 0.88–400 µM (Table 1). Complex formation was significantly slower and less efficient at low tartrate concentrations, and became apparently independent on tartrate concentration at higher values (Fig. 4A and 4B). As a matter of fact, visible spectra show significant changes in the blue-edge region at tartrate concentration higher than 100 µM (Fig. S4). This suggests that a different complex was formed under those conditions. Therefore, the optimal concentration range for tartrate was 20–100 µM (Fig. 4B and Table 1).

Figs. 1B, 2B, 3B, and 4B also compare absorbance values at 710 and 890 nm. The dependence on reagent concentration is similar at both wavelengths, but the signal is significantly higher at 890 nm. We observed that the signal-to-noise ratio at 890 nm is lower than that at 710 nm (3.5·10^2^ with respect to 1·10^4^, see Fig. S5), probably due to the fact that the maximal detection wavelength of our spectrophotometer is 900 nm. Therefore, we selected a detection wavelength of 850 nm to obtain a high signal (>85% with respect to 700 nm, Fig. S5) with a good signal-to-noise ratio (2·10^3^, Fig. S5).

In conclusion, based on the experiments described above we decided to perform absorbance measurements at 850 nm and to prepare a coloring solution with the following composition: 125 mM H_2_SO_4_, 0.50 mM ammonium-molybdate, 10 mM ascorbic acid and 40 µM tartrate.

### Hydrolysis of ATP and color stability

**Figure 5:**
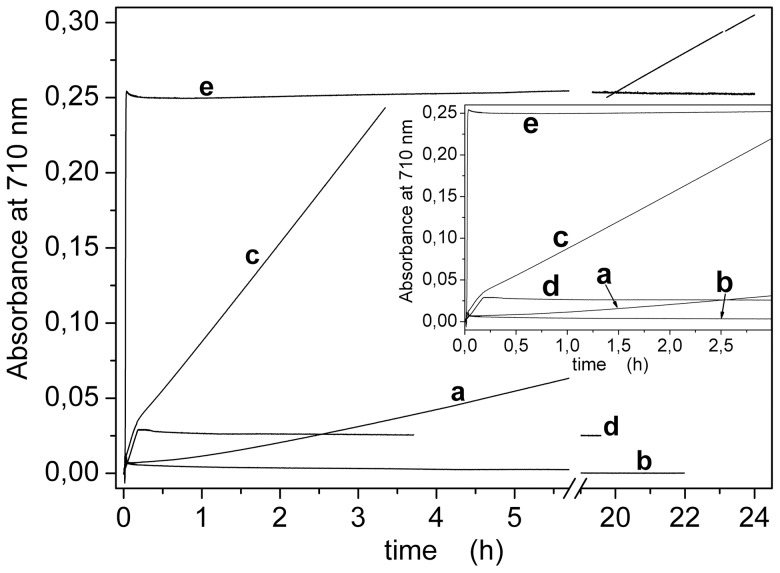
Color stability. Color development curves monitored for 24 hours under different experimental conditions: 1 mM ATP (a); 1 mM ATP followed by the addition of 3% citrate (b); 5 mM ATP (c); 5 mM ATP followed by the addition of 0.2% citrate (d). The curve obtained in the presence of 15 nmol Pi is also reported for comparison (e). The inset shows the same curves within a smaller time interval (3 hours). From the slope of the curves acquired in the presence of 1 and 5 mM ATP (a and c, respectively), the rate of ATP hydrolysis can be estimated (see text).

It is known that ATP undergoes hydrolysis in acid conditions. This might be a problem for activity measurements on ATPase enzymes, since acid-released-Pi may be produced, causing a chemical interference [23]. For this reason, we quantified the amount of Pi released by ATP under the acid conditions of the coloring solution (Fig. 5).

Results show that after 1 hour the absorbance increased of about 3% using 1 mM ATP and 30% using 5 mM ATP. Percentages are expressed with respect to the stationary absorbance value attained in the presence of 15 nmol Pi (Fig. 5). The corresponding amounts of released Pi were 0.45 nmol and 4.5 nmol, respectively, and the rates of ATP hydrolysis 0.45 nmol/h and 4.5 nmol/h.

When determining the hydrolytic activity of ATPases, acid ATP hydrolysis occurs during the time elapsed between addition of the aliquot to the coloring solution and absorbance measurement (“elapsed time”). Obviously, the interference due to nascent Pi can be minimized using 1 mM ATP, a concentration that is largely sufficient for the activity measurements (see below). Nevertheless, the contribution of the acid-released-Pi should be subtracted from the total Pi using a blank sample (see Materials and Methods). This correction is acceptable for short elapsed times (Fig. 5). For long elapsed times, addition of sodium citrate might be helpful. In fact, sodium citrate [23] or a citrate-arsenite mixture [24] act as stabilizing agents by chelating molybdenum, thus preventing the detection of nascent inorganic phosphate. This is also shown in Fig. 5 where, in the presence of citrate, the absorbance level remains stable for several hours.

### Calibration curve

**Figure 6:**
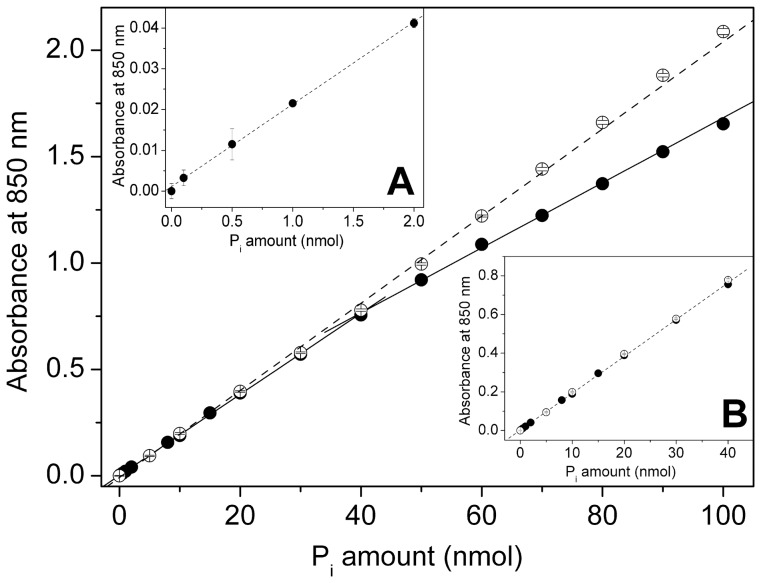
Calibration curves. Calibration curves determined in the absence (○) or in the presence (•) of 0.2 % citrate. The parameters obtained from the linear fitting of the experimental data (plain or dashed lines) are summarized in Table 2. The two insets show the same curves for different concentration ranges. A significant absorbance can be measured even in the presence of 0.1 nmol Pi (inset A), whereas the presence of citrate has no effect below 40 nmol Pi (inset B). Error bars represent the SEM of at least three independent measurements and, where not visible, are masked by symbols. Elapsed time: 30 minutes.

We then determined the calibration curve at 850 nm and the linearity range extension for the method. It is evident from Fig. 6 that, using the optimized experimental conditions, this method allows the determination of sub-nanomoles of Pi.

In the absence of sodium citrate, the calibration curve remained perfectly linear up to 100 nmol Pi (Fig. 6 and Table 2), and the estimated molar extinction coefficient was about 2.05·10^5^ M^-1^cm^-1^. However, in the presence of citrate two regions are distinguishable, below and above 40 nmol Pi. Below 40 nmol Pi linearity was still excellent (Table 2), with a molar extinction coefficient in agreement with that determined without citrate (1.90·10^5^ M^-1^cm^-1^). Above 40 nmol a linear trend is still observed, but the slope of the calibration curve slightly decreases (Table 2), as if a different chemical species may be present in solution (ε  =  1.5·10^5^ M^-1^cm^-1^). However, the presence of citrate has no effect below 40 nmol Pi (Fig. 6, inset B). Representative visible spectra were reported in Fig. S6.

**Table 2:** Calibration curves.

Sodium citrate	Pi concentration range	Intercept	Slope	R	Estimated ε
	(nmol)		(nmol ^-1^)		(M^-1^cm^-1^)
–	0–100	–0.008(1)	0.02048(5)	0,99953	2.05·10^5^
+	0–2	0.001(1)	0.0202(9)	0.99947	2.0·10^5^
+	0–40	0.0020(5)	0.01905(6)	0.99983	1.90·10^5^
+	40–100	0.155(8)	0.0153(1)	0.99905	1.5·10^5^

Fitting parameters for experimental data reported in Fig. 6. The uncertainty on the last digit is indicated between parenthesis.

Considering that a detectable signal was already present with 0.1 nmol Pi (Fig.6, inset A), with a very good correlation also below 2 nmol Pi (Table 2), the range of linearity for the present method is 0.1–40 or 0.1–100 nmol Pi, with or without citrate, respectively.

### ATPase activity determination

To demonstrate the validity of this method, the hydrolytic activities of different ATPases were determined. Activity measurements were carried out on native (SERCA and Na,K-ATPase) and recombinant proteins (WT and D351N mutant SERCA) at 37°C. Control experiments were performed in the presence of specific inhibitors, i.e. TG for SERCA [25], [26] or ouabain for the Na,K-ATPase [27].

### Native SERCA

**Figure 7:**
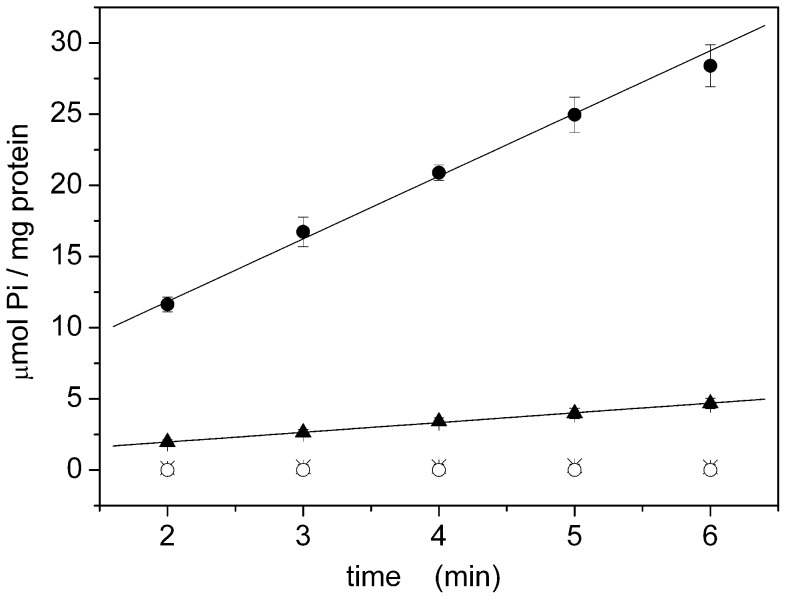
Hydrolytic activity of native SERCA. Enzymatic activity of native SERCA determined in the presence of 10 µM free calcium (•, ▴, X) and in the presence (•, X) or in the absence (▴) of 2 µM A23187. As control experiments, Pi release was also quantified in the absence of calcium ions (○) or in the presence of 1 µM TG (X). Solid lines represent the linear fitting (Y = A+BX) of the experimental data: A =  3 ± 1 µmol Pi/mg; B =  4.4 ± 0.3 µmol Pi/(mg·min) (+A23187); A =  0.6 ± 0.3 µmol Pi/mg; B =  0.68 ± 0.08 µmol Pi/(mg·min) (-A23187). Error bars represent the standard deviations of three measurements. Elapsed time: 30 minutes.

We first examined SERCA contained in native vesicles (microsomes) and isolated from rabbit skeletal muscle [19]. The measured activity was 4.3 ± 0.1 µmol Pi / (min·mg) (Fig. 7 and Table 3) expressed as average of three independent measurements ±SEM. SERCA content corresponds approximately to 50% of the total protein [1], therefore SERCA hydrolytic activity is about 8–9 µmol Pi / (min·mg). Considering a molecular mass of 110 kDa for the protein [28], this value corresponds to a turnover rate of about 15–17 s^-1^ (Table 3).

**Table 3:** Enzyme activity.

Enzyme	A23187	Hydrolytic activity(µmol Pi / (mg·min))		Turnover rate(s^-1^)
		**Total protein**	**SERCA only**	
Native SERCA	present	4.3±0.1	∼8.5	15.6
	absent	0.68±0.08	∼1.4	2.6
Native Na,K-ATPase	---	11.8±0.7	---	30
Recombinant SERCA (WT)	present	0.28±0.04	∼9	16.5
	absent	0.15±0.02	∼0.5	0.9

Hydrolytic activity values, with the corresponding turnover rates, determined for native SERCA, native Na,K-ATPase and recombinant SERCA (WT) at 37°C. In the case of SERCA, the presence or absence of 2 µM calcium ionophore (A23187) is specified, and the activity values are expressed with respect to the total protein or to SERCA only (see text).

Enzyme activity was fully blocked by 1 µM TG and by removing calcium ions from the solution following addition of 2 mM EGTA (Fig. 7). This confirms the absence of other Ca-dependent phosphatases in the preparation. Moreover, in the absence of the calcium ionophore A23187 the measured hydrolytic activity was significantly lower (0.68 ± 0.08 µmol Pi / (min·mg); Fig. 7 and Table 3). Hence, the absence of ionophore causes a rise of lumenal Ca^2+^ concentration to mM values with rapid saturation of the vesicles by Ca^2+^ and consequent “back-inhibition” of the pump [29]. This result is also an indirect confirmation that microsomes are not leaky to Ca^2+^ ions.

### Native Na,K-ATPase

**Figure 8:**
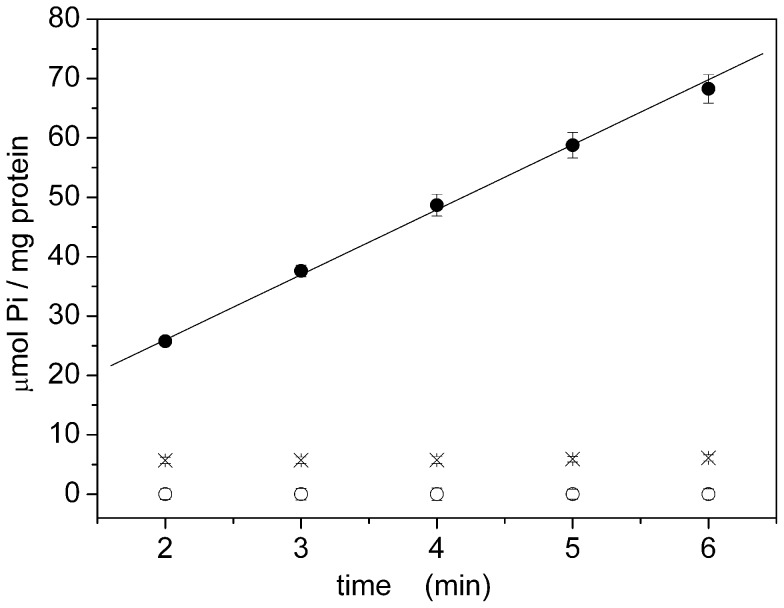
Hydrolytic activity of native Na,K-ATPase. Enzymatic activity of native Na,K-ATPase determined in the presence of sodium ions (•, X). Background activity in the absence of sodium ions (○) or in the presence of 50 µM ouabain (X) are also shown. The solid line represent the linear fitting (Y = A+BX) of the experimental data: A =  4 ± 1 µmol Pi/mg; B =  10.9 ± 0.5 µmol Pi/(mg·min). Error bars represent the standard deviations of three measurements. Elapsed time: 30 minutes.

Similar measurements were performed on purified (open) membrane fragments containing the Na,K-ATPase (∼99% Na,K-ATPase). An activity value of 11.8 ± 0.7 µmol Pi / (min·mg) (average of three independent measurements ±SEM) was determined (Fig. 8 and Table 3). Here again, Pi production by the enzyme was fully blocked by 50 µM ouabain and by eliminating sodium ions from the buffer solution (Fig. 8). This indicates that the measured activity is entirely ascribable to the Na,K-ATPase. With a molecular mass of 155 kDa [3], the related turnover rate is about 30 s^-1^ (Table 3).

### Recombinant SERCA (WT and D351N)

We then extended this method to recombinant ATPases, that are notoriously produced at significantly lower concentration with respect to native enzymes (see Materials and Methods).

**Figure 9:**
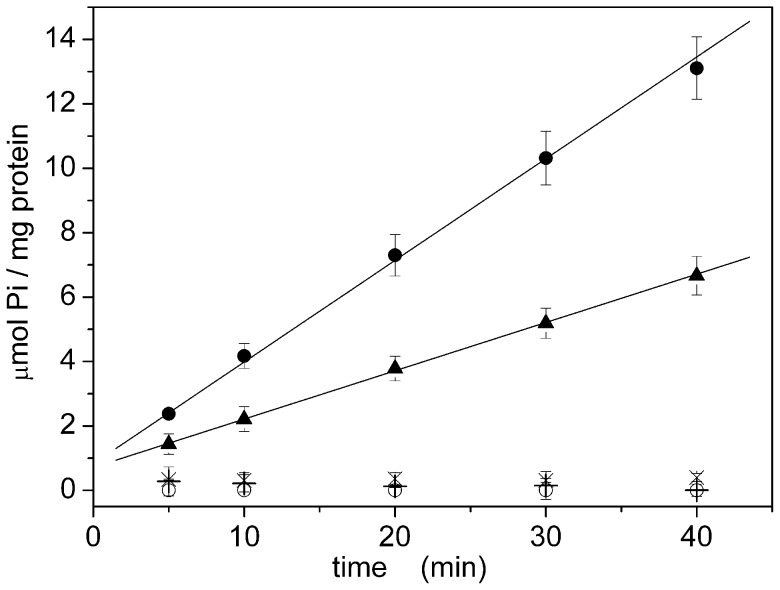
Hydrolytic activity for recombinant SERCA. Enzymatic activity for WT recombinant SERCA (•, ▴, ○, X) and the D351N mutant (┼). Activity was determined in the presence of 10 µM free calcium (•, ▴, X, ┼) and in the presence (•, X, ┼) or in the absence (▴) of 2 µM A23187. Pi release was also quantified in the absence of calcium ions (○) or in the presence of 1 µM TG (X). Solid lines represent the linear fitting of the experimental data: A =  0.8 ± 0.2 µmol Pi/mg; B =  0.32 ± 0.02 µmol Pi/(mg·min) (+A23187); A =  0.7 ± 0.3 µmol Pi/mg; B =  0.15 ± 0.02 µmol Pi/(mg·min) (-A23187). Error bars represent the standard deviations of three measurements. Elapsed time: 90 minutes.

Recombinant (WT) SERCA exhibited a hydrolytic activity of 0.28±0.04 µmol Pi / (min·mg), considering the total protein (Fig. 9 and Table 3). This value increases to 9.3±1.3 µmol Pi / (min·mg) if we consider the effective SERCA content (3% for this preparation), which is in good agreement with the value determined for the native protein (Table 3). It is worth noting that, due to the low protein concentration, a longer enzymatic reaction time is necessary to detect a Pi concentration with a good signal-to-noise ratio. Therefore, measurements on recombinant SERCA were performed over a time period of 40 minutes (Fig. 9), whereas few minutes were sufficient for measurements on native proteins (Figs. 7 and 8). The longer time interval determined a longer elapsed time, and required the use of citrate.

Hydrolytic activity of recombinant SERCA was fully abolished by 1 µM TG or by elimination of calcium ions from the buffer solution (Fig. 9). In the absence of calcium ionophore the activity is approximately halved (0.15±0.02 µmol Pi / (min·mg), Fig. 9 and Table 3). This demonstrates that “recombinant” microsomes are not leaky to calcium ions, as observed for “native” microsomes.

A further control was carried out by using the catalytically inactive D351N mutant. This mutant can bind calcium ions but is unable to hydrolyze ATP [29], due to mutation of the conserved Asp residue (D351) with an Asn. Accordingly, we found that no inorganic phosphate was produced by the D351N mutant (Fig. 9).

## Discussion

The detection of small amounts (nanomoles) of inorganic phosphate has a broad interest that spans from environmental to biochemical fields [6], [7], [17], [18], [23], [30]–[32]. The present method optimizes an experimental protocol usually employed for environmental analysis to allow its use with phosphatase enzymes, such as ATPases. Our method introduces significant improvements to well-established experimental assays, which are currently employed for ATPase activity measurements.

### Comparison with other methods

Classical protocols for ATPase activity determination include both coupled-enzyme-based and molybdenum-based methods. An example of the former method is that introduced by Schwartz et al. [33], that makes use of the enzymes pyruvate kinase (PK) and lactate dehydrogenase (LDH) and the reagents phosphoenolpyruvate and NADH. In this method, the released phosphate enters a cascade reaction involving firstly PK, that regenerates ATP thanks to the dephosphorylation of phosphoenolpyruvate. The pyruvate so produced, is converted to lactate by LDH with the simultaneous oxidation of NADH. By monitoring the absorbance at 340 nm with a spectrophotometer, the rate of absorbance decrease (correlated to NADH oxidation) can be determined. This rate is proportional to the rate of phosphate release by the ATPase, since the reaction catalyzed by PK and LDH are much faster.

The enzymatic method of Schwartz et al. [33] is a rapid and easy test that can be performed on ATPases, as reported in the literature [34], [35]. On the other hand, PK and LDH enzymes usually need a careful storage (e.g. at -20°C) and their complete functionality has to be checked periodically. Moreover, in case of inhibition studies of ATPases, appropriate control experiments must be carried out to exclude any interference of the inhibitor with the reactions catalyzed by PK and LDH. Obviously, a non-enzymatic method does not require such controls. Incidentally, this also makes a non-enzymatic test more suitable for a possible employment in a high throughput device for automatic drug screening.

On the other hand, a classical experimental molybdenum-based protocol used with ATPases is the Lanzetta method [23]. This method allows the determination of nanomoles of Pi using malachite green as a peculiar agent, that produces a complex with the phospho-molybdate compound [36]. A detergent is also necessary to stabilize the final complex, originally sterox [23] but also tween20 [32], [36], [37] or TritonX [31] have been used as valid alternatives. In the Lanzetta method the coloring solution needs to be mixed for at least 20 minutes and filtrated before use. The authors also suggested the use of citrate (final concentration ∼3%) to block development of nascent Pi due to the acid environment and to the catalytic effect of molybdenum [23]. Under the typical experimental conditions of the Lanzetta method, the ATP acid hydrolysis rate is estimated to be 2.70 nmol Pi/h in the presence of 26 µM ATP [23]. Color fully develops in 30 minutes and solutions have a stability of about 4 hours [23]. We used the Lanzetta method in our previous studies, confirming all these experimental findings [30], [38], [39].

The method based on potassium-antimony (III) oxide tartrate, described in the present article, has never been applied to ATPase enzymes, to our knowledge. This method appears to be as sensitive as the Lanzetta test (Fig. 6), but introduces some significant experimental advantages:

- 1.The method has a wide linearity range, from 0.1 nmol to 100 nmol of Pi, if citrate is not employed. The linearity range reduces to 0.1-40 nmol in the presence of citrate (Fig. 6 and Table 2);
- 2.There is no need to use a detergent nor to perform extensive mixing and filtrate the coloring solution before use. The preparation of the coloring solution is therefore easy and fast;
- 3.Color develops very rapidly (about 2-3 min, e.g. Fig. 1A) and is stable for several hours (Fig. 5);
- 4.Using our method, the ATP acid hydrolysis rate is significantly lower (0.45 nmol/h in the presence 1 mM ATP, Fig. 5) with respect to the Lanzetta method (2.70 nmol/h in the presence of 26 µM ATP [23]). Hence, addition of citrate is not necessary for short elapsed times (as in the case of native proteins);
- 5.When citrate becomes necessary, a final concentration of 0.2% is sufficient to stabilize color (Fig. 5), with respect to ∼3% as reported by [23]. This significantly reduces the consumption of this reagent.

It is worth noting that the molybdenum blue produced in this manner has a maximum of absorption at 850 nm (Fig. S6), indicating that a different reduced species is formed with respect to the Lanzetta method (maximum wavelength 660 nm).

For all these reasons, this method turns out to be a new, fast, stable, reliable and sensitive system to detect nanomoles of Pi released by ATPases.

### Application to ATPase enzymes

Our method was successfully applied to both native and recombinant ATPases. Whereas native ATPases can be isolated in high concentration, at present recombinant proteins can only be produced at low yield. To limit the use of recombinant protein, we need a sensitive experimental method, which is able to detect low amounts of analyte with a good signal-to-noise ratio.

Using the present method we determined the hydrolytic activity of both native and recombinant ATPases, and we obtained experimental results (i.e. activities and turnover rates) in agreement with those reported in the literature. In particular, knowing the molecular mass of the enzyme we calculated turnover rates from the measured hydrolytic activities (Table 3). We found that SERCA (both native and recombinant) has a turnover rate of 15–17 s^-1^, whereas a rate of 30 s^-1^ was obtained in the case of native Na,K-ATPase. The SERCA turnover rate agrees with that reported in the literature at the same temperature (37°C) [40]. The turnover rate calculated for Na,K-ATPase is lower with respect to the values reported by Clarke and colleagues [41], [42], probably because some of the Na,K-ATPase molecules are denaturated or for some reason inactive in our preparation. However, lower values for the Na,K-ATPase activity (and hence the turnover rate) at 37°C, similar to that obtained in our measurements, were also reported [43], [44].

In the case of SERCA, the turnover is slower in the absence of the calcium ionophore A23187, due to excessive Ca^2+^ accumulation into the microsomes. By comparing the turnover rates in the absence of A23187 for native and recombinant SERCA (2.6 s^-1^ vs 0.9 s^-1^; Table 3), it appears that ATP-dependent Ca^2+^ accumulation into “native” microsomes is about 3 times faster with respect to “recombinant” microsomes.

### Conclusions

A sensitive, fast, sound, reliable and experimentally simple method was developed to determine hydrolytic activity of phosphatases. The method can be easily applied to ATPase enzymes, membrane proteins with many important physiological roles that are emerging as potential drug targets [45], [46]. The measurement of ATPase hydrolytic activity is necessary to test enzyme functionality, but it is also useful to reveal possible inhibitory effects of molecules that interfere with the hydrolytic process. Therefore, this method may be valuable in biochemical and biomedical investigations of ATPase enzymes, in combination with more specific tests, as well as in high throughput drug screening.

## Supporting Information

Figure S1 **Dependence on H_2_SO_4_ concentration.** Visible spectra acquired for different H_2_SO_4_ concentrations as indicated in the legend.
(TIF)

Figure S2 **Dependence on ammonium heptamolybdate concentration.** Visible spectra acquired for different ammonium heptamolybdate concentrations (see legend).
(TIF)

Figure S3 **Dependence on ascorbic acid concentration.** Visible spectra corresponding to different ascorbic acid concentrations according to the legend.
(TIF)

Figure S4 **Dependence on potassium-antimony (III) oxide tartrate concentration.** Visible spectra acquired for different tartrate concentrations (see legend).
(TIF)

Figure S5 **Signal-to-noise ratio.** (A) Representative stationary absorbance levels determined in the presence of 20 nmol P_i_ at different wavelengths (see legends). (B) Dependence of the absorbance (•) and of the signal-to-noise ratio (○) on the working wavelength. Absorbance was normalized with respect to the value attained at 700 nm. The signal-to-noise ratio at each wavelength is given by the ratio of the average absorbance to the corresponding standard deviation. Averages were calculated over a time period of 4 minutes for each trace in panel A. The normalized absorbance and signal-to-noise ratio at the selected wavelength of 850 nm are indicated by the dashed lines.
(TIF)

Figure S6 **Dependence on phosphate concentration.** Visible spectra acquired for different phosphate concentrations (see legend). The coloring solution had the composition determined after the optimization procedure (see text).
(TIF)
