# Supplementary figures and images for: A Method to Measure Hydrolytic Activity of Adenosinetriphosphatases (ATPases)
Source: PLoS One. 2013 Mar 5;8(3):e58615. doi: 10.1371/journal.pone.0058615 (PMC3589382; doi:10.1371/journal.pone.0058615)

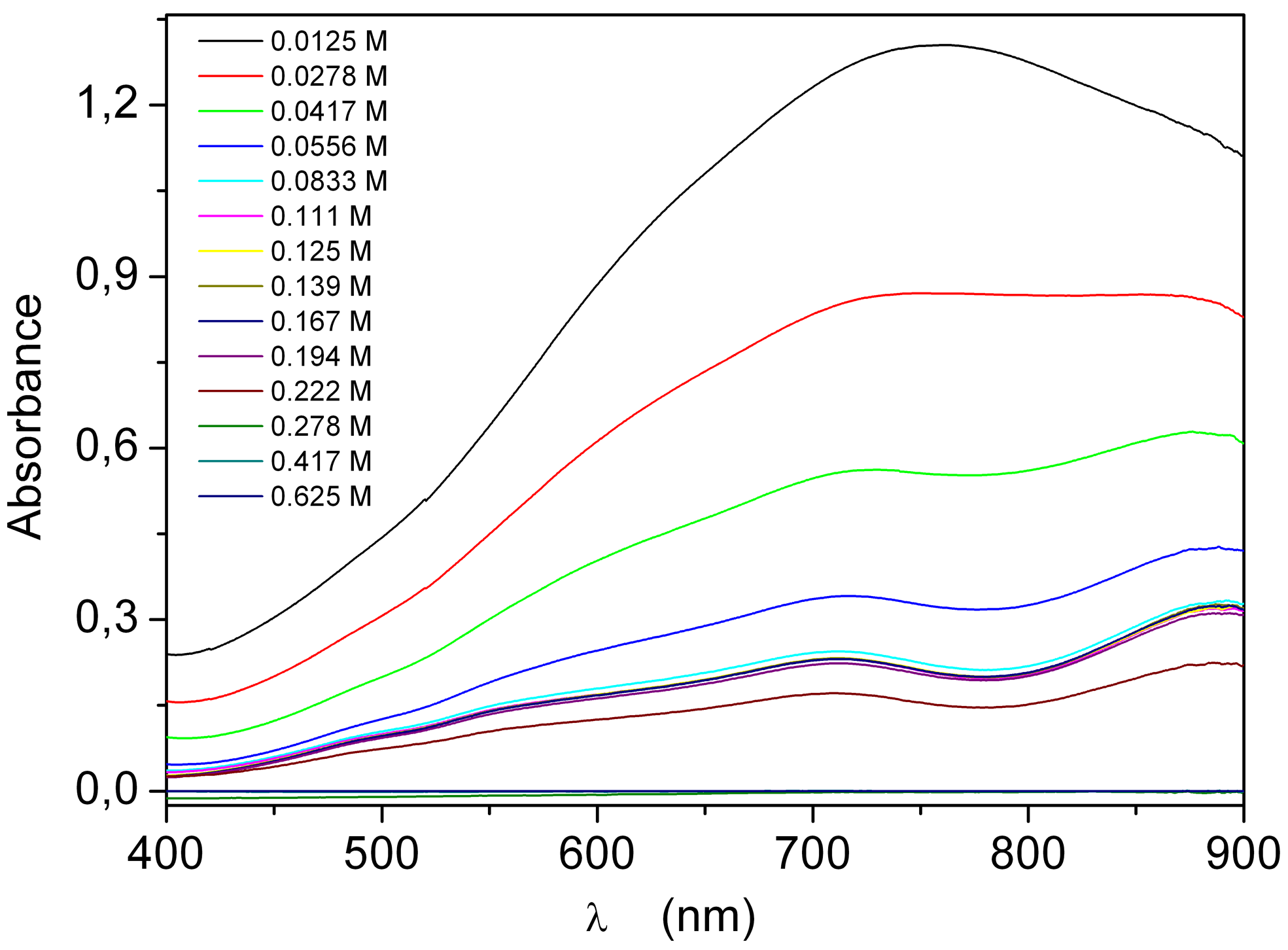

Supplement: Figure S1 — Dependence on H2SO4 concentration. Visible spectra acquired for different H2SO4 concentrations as indicated in the legend. (TIF) [file pone.0058615.s001.tif]

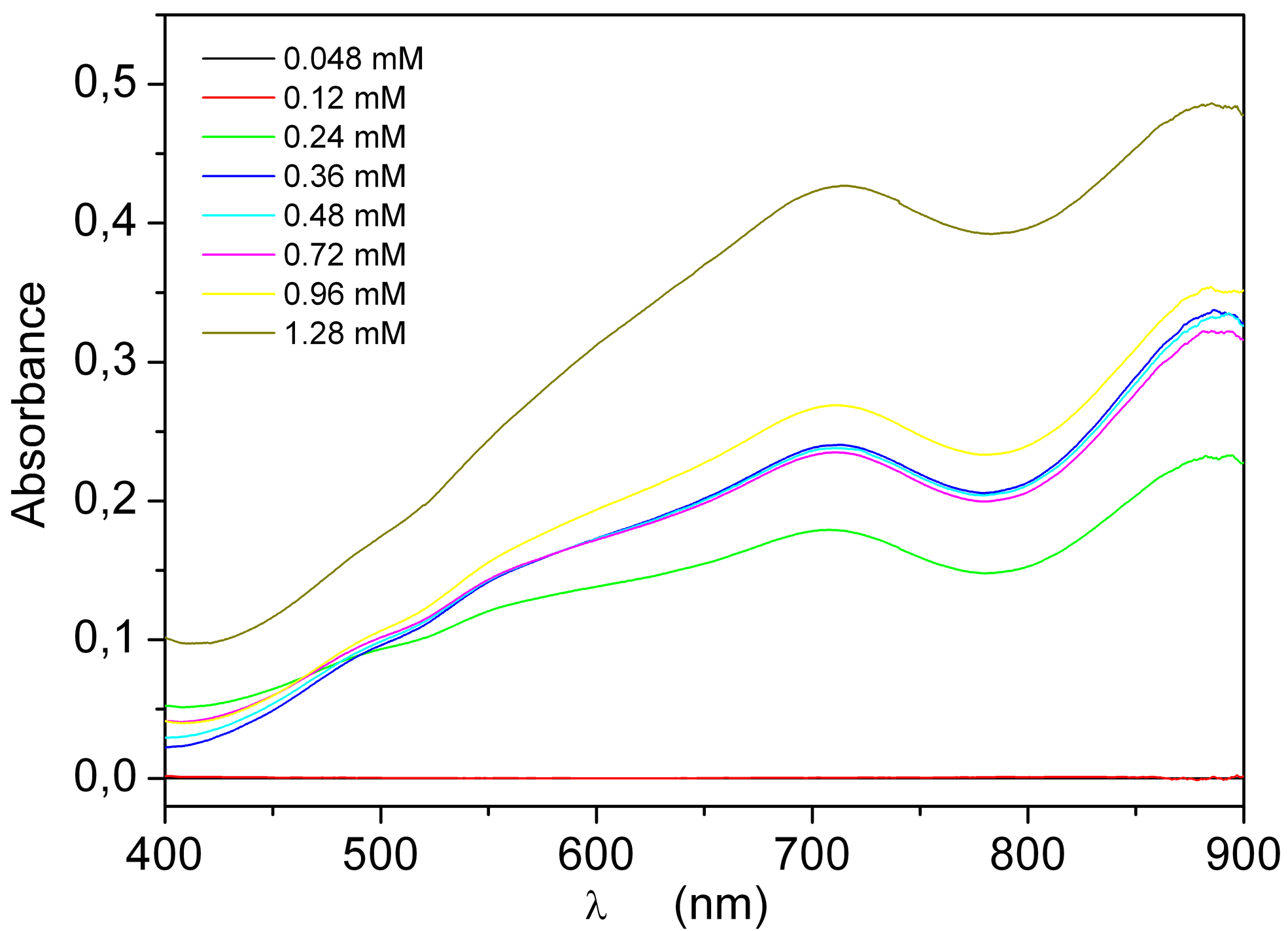

Supplement: Figure S2 — Dependence on ammonium heptamolybdate concentration. Visible spectra acquired for different ammonium heptamolybdate concentrations (see legend). (TIF) [file pone.0058615.s002.tif]

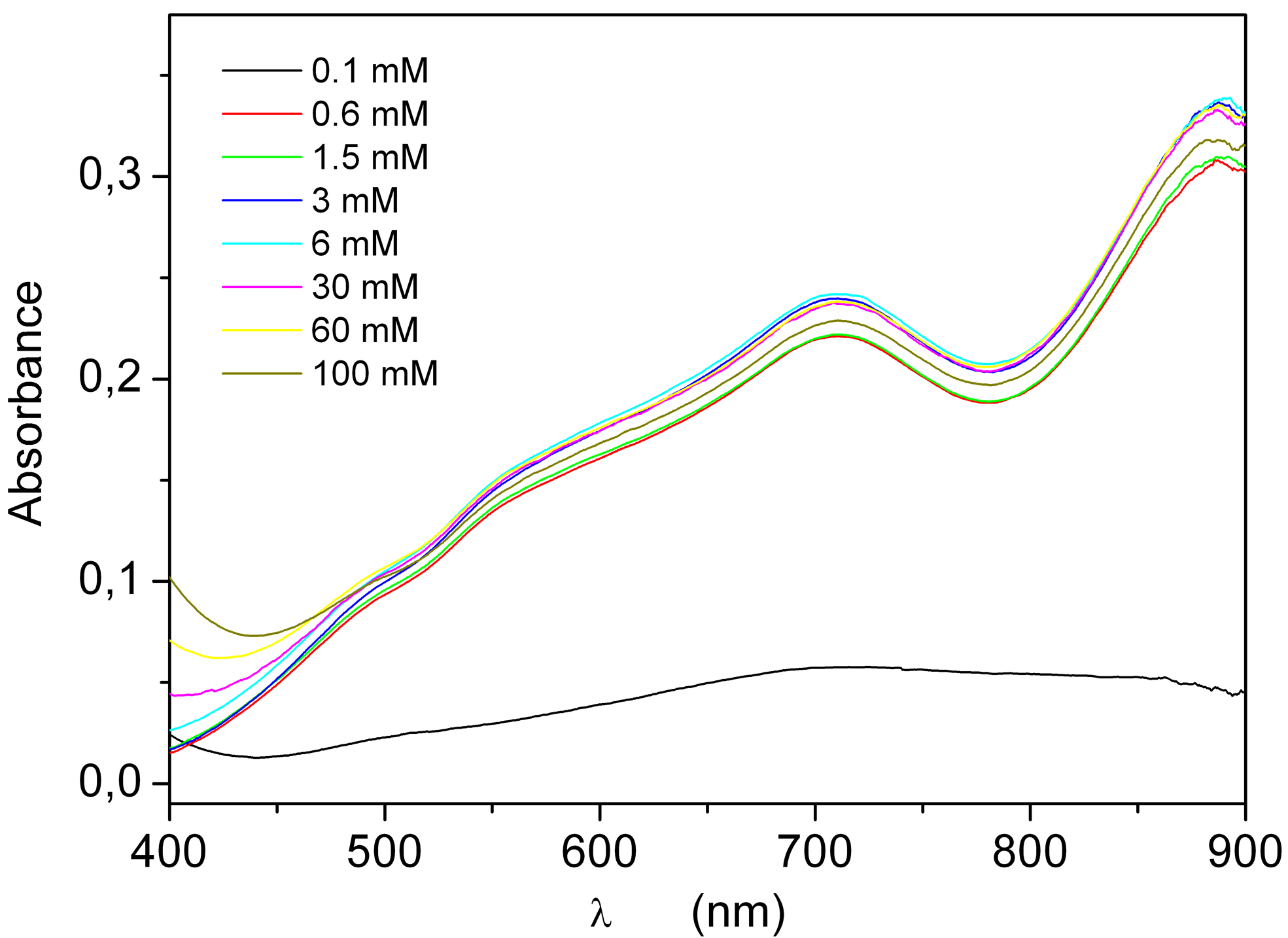

Supplement: Figure S3 — Dependence on ascorbic acid concentration. Visible spectra corresponding to different ascorbic acid concentrations according to the legend. (TIF) [file pone.0058615.s003.tif]

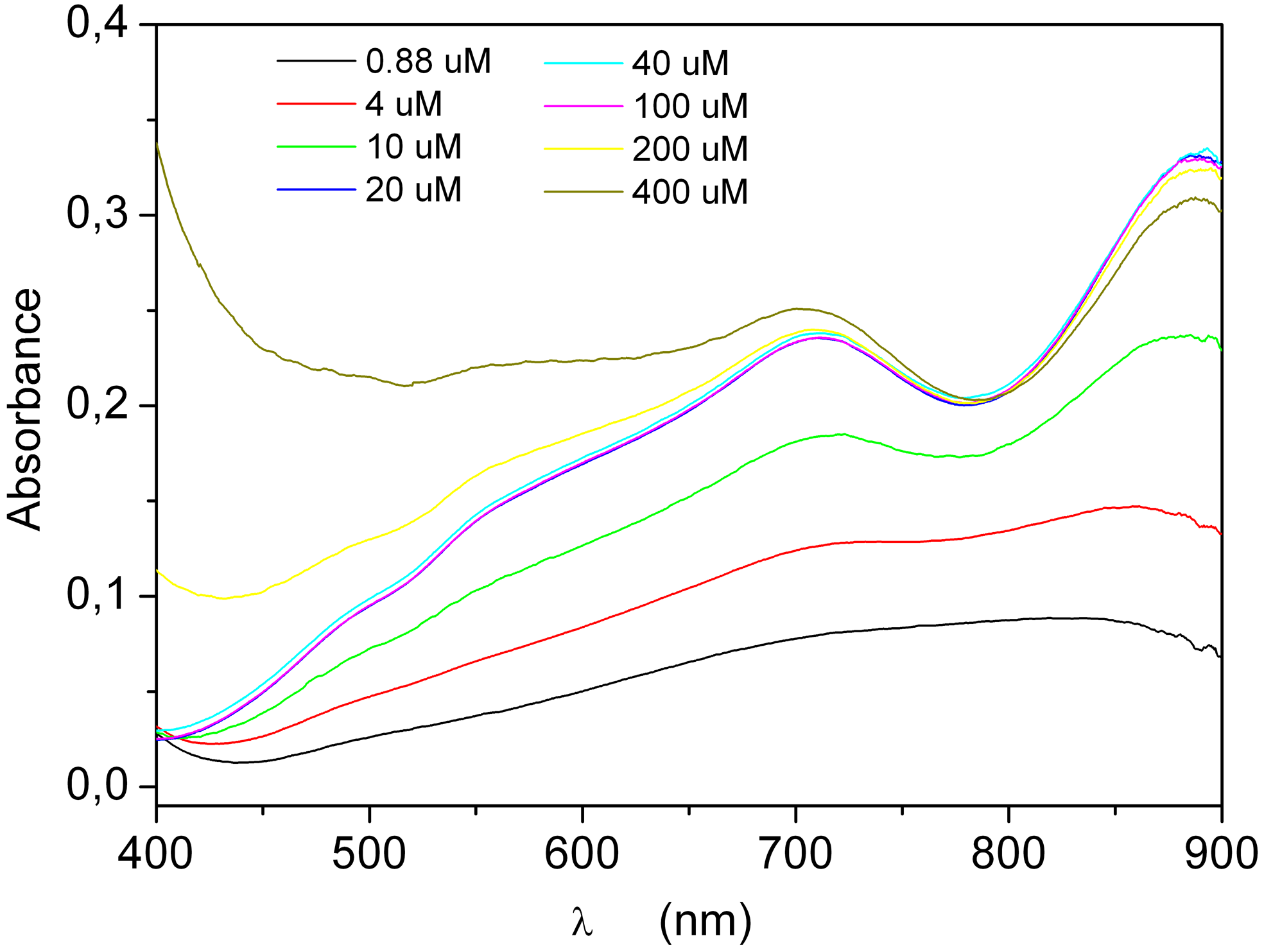

Supplement: Figure S4 — Dependence on potassium-antimony (III) oxide tartrate concentration. Visible spectra acquired for different tartrate concentrations (see legend). (TIF) [file pone.0058615.s004.tif]

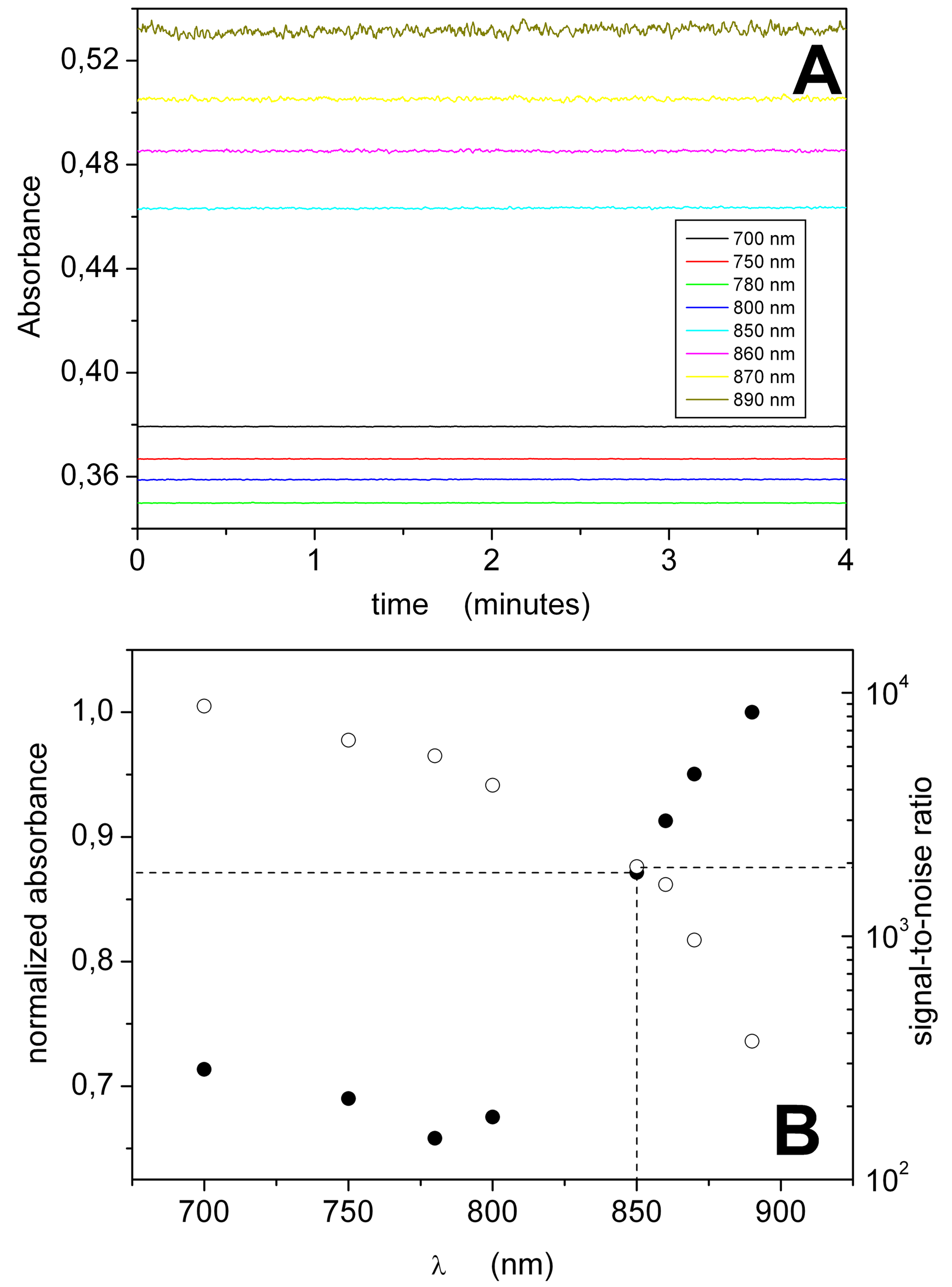

Supplement: Figure S5 — Signal-to-noise ratio. (A) Representative stationary absorbance levels determined in the presence of 20 nmol Pi at different wavelengths (see legends). (B) Dependence of the absorbance (•) and of the signal-to-noise ratio (○) on the working wavelength. Absorbance was normalized with respect to the value attained at 700 nm. The signal-to-noise ratio at each wavelength is given by the ratio of the average absorbance to the corresponding standard deviation. Averages were calculated over a time period of 4 minutes for each trace in panel A. The normalized absorbance and signal-to-noise ratio at the selected wavelength of 850 nm are indicated by the dashed lines. (TIF) [file pone.0058615.s005.tif]

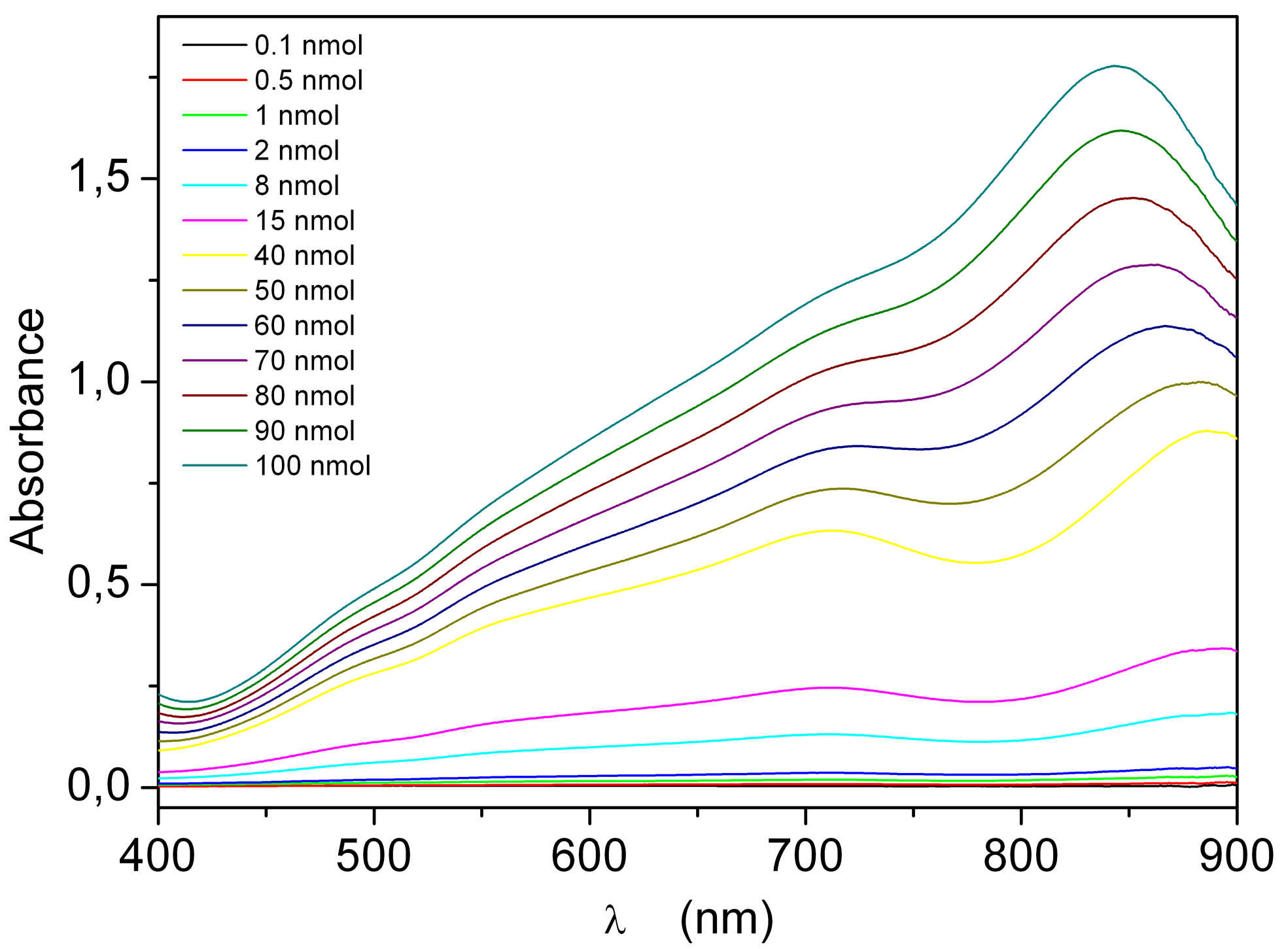

Supplement: Figure S6 — Dependence on phosphate concentration. Visible spectra acquired for different phosphate concentrations (see legend). The coloring solution had the composition determined after the optimization procedure (see text). (TIF) [file pone.0058615.s006.tif]
